# Submaximal Oxygen Deficit During Incremental Treadmill Exercise in Elite Youth Female Handball Players

**DOI:** 10.3390/sports13080252

**Published:** 2025-07-31

**Authors:** Bettina Béres, István Györe, Annamária Zsákai, Tamas Dobronyi, Peter Bakonyi, Tamás Szabó

**Affiliations:** 1 Department of Health Sciences and Sport Medicine, Hungarian University of Sports Science, 1123 Budapest, Hungary; dr.gyoreistvan@gmail.com; 2 Sport Sciences and Diagnostic Research Centre, Hungarian Handball Federation, 1103 Budapest, Hungary; annamaria.zsakai@ttk.elte.hu (A.Z.); dobronyi.tamas@gmail.com (T.D.); bakonyi.peti@gmail.com (P.B.); szabo.tamas@mksz.hu (T.S.); 3 Department of Biological Anthropology, Eotvos Lorand University, 1117 Budapest, Hungary

**Keywords:** handball, oxygen deficit, spiroergometry, body composition, PCA, cluster analysis

## Abstract

Laboratory-based assessment of cardiorespiratory function is a widely applied method in sports science. Most performance evaluations focus on oxygen uptake parameters. Despite the well-established concept of oxygen deficit introduced by Hill in the 1920s, relatively few studies have examined its behavior during submaximal exercise, with limited exploration of deficit dynamics. The present study aimed to analyze the behavior of oxygen deficit in young female handball players (N = 42, age: 15.4 ± 1.3 years) during graded exercise. Oxygen deficit was estimated using the American College of Sports Medicine (ACSM) algorithm, restricted to subanaerobic threshold segments of a quasi-ramp exercise protocol. Cardiorespiratory parameters were measured with the spiroergometry test on treadmills, and body composition was assessed via Dual Energy X-ray Absorptiometry (DEXA). Cluster and principal component analyzes revealed two distinct athlete profiles with statistically significant differences in both morphological and physiological traits. Cluster 2 showed significantly higher relative VO_2_ peak (51.43 ± 3.70 vs. 45.70 ± 2.87 mL·kg^−1^·min^−1^; *p* < 0.001; Cohen’s d = 1.76), yet also exhibited a greater oxygen deficit per kilogram (39.03 ± 16.71 vs. 32.56 ± 14.33 mL·kg^−1^; *p* = 0.018; d = 0.80). Cluster 1 had higher absolute body mass (69.67 ± 8.13 vs. 59.66 ± 6.81 kg; *p* < 0.001), skeletal muscle mass (*p* < 0.001), and fat mass (*p* < 0.001), indicating that body composition strongly influenced oxygen deficit values. The observed differences in oxygen deficit profiles suggest a strong influence of genetic predispositions, particularly in cardiovascular and muscular oxygen utilization capacity. Age also emerged as a critical factor in determining the potential for adaptation. Oxygen deficit during submaximal exercise appears to be a multifactorial phenomenon shaped by structural and physiological traits. While certain influencing factors can be modified through training, others especially those of genetic origin pose inherent limitations. Early development of cardiorespiratory capacity may offer the most effective strategy for long-term optimization.

## 1. Introduction

Performance in team sports is determined by a complex interplay of technical, tactical, and physical attributes. Athletes are required to demonstrate high levels of speed, strength, agility, and strategic execution to meet the demands of high-intensity competition [1]. In handball, these requirements are especially pronounced due to the dynamic nature of the game, which involves frequent transitions, explosive movements, and variable intensity. The interplay of energy systems during handball matches reflects the continuous shift between aerobic and anaerobic metabolism depending on the intensity of the activity. Although the speed of play has significantly increased in recent years, the importance of a well-developed aerobic base remains indispensable for sustaining performance and facilitating recovery [2].

The interaction between aerobic and anaerobic energy systems remains a central topic in exercise physiology, particularly concerning performance optimization and recovery mechanisms. The concept of maximal oxygen uptake (VO_2_ max) was first introduced by Hill in the 1920s, who highlighted the temporal discrepancy between oxygen demand and delivery during exercise. He proposed that the shortfall in oxygen supply termed “oxygen deficit” was initially compensated by anaerobic metabolism, which leads to lactic acid accumulation. He also introduced the concept of “oxygen debt,” describing elevated post-exercise oxygen consumption during recovery, and laid the foundations of modern exercise physiology by modeling oxygen kinetics in response to workload changes [3].

Oxygen deficit is commonly used as an indicator of aerobic power and metabolic stress. It is defined as the integral over time of the difference between oxygen demand and oxygen uptake during exercise [4]. Training-induced reductions in oxygen deficit have been associated with improved endurance capacity and delayed onset of exhaustion in high-intensity efforts [5]. This makes oxygen deficit a valuable parameter for monitoring adaptations to endurance training.

Incremental exercise testing, particularly in controlled laboratory settings, enables the assessment of physiological responses across various intensity domains [6]. During such testing, increasing workloads induce progressive changes in oxygen uptake, reflecting shifts between aerobic and anaerobic metabolic pathways [7]. Whipp and Wasserman [8] described the biphasic nature of oxygen uptake kinetics during incremental exercise: at submaximal intensities, VO_2_ reaches a steady state, minimizing oxygen deficit, while at suprathreshold intensities, delayed VO_2_ kinetics result in cumulative oxygen deficit and elevated lactate production. This phenomenon is further complicated by the VO_2_ “slow component,” observed during sustained high-intensity exercise [9].

Estimating oxygen deficit is more straightforward at submaximal workloads, where oxygen uptake stabilizes and reflects demand. However, at high intensities, this estimation becomes more complex due to nonlinear relationships and declining mechanical efficiency [10]. Moreover, interruptions to exercise before achieving VO_2_ max can hinder the accurate determination of oxygen demand [11]. The development of the oxygen deficit during exercise is modulated by a range of physiological and morphological determinants, such as cardiorespiratory efficiency, pulmonary function, oxygen transport mechanisms, muscle fibre typology, and the metabolic capacities of both oxidative and glycolytic pathways [12].

Among these, body composition plays a pivotal role in determining aerobic capacity. Previous studies have demonstrated strong correlations between oxygen uptake and anthropometric parameters such as body mass, height, chest circumference, and fat-free mass [13]. In adolescent athletes, individual differences in growth and maturation further impact oxygen transport and utilization. Greater muscle mass is typically associated with improved VO_2_ max and endurance performance, while higher body fat percentages negatively affect aerobic efficiency and post-exercise recovery [14,15,16]. Moreover, in youth populations, body dimensions and somatotype classifications have been shown to influence ventilatory parameters and overall cardiorespiratory fitness [17,18].

In handball, players typically cover approximately 5 km during a match, with high-intensity efforts accounting for around 2.5% of total distance. The frequent changes in pace, averaging 700 intensity transitions per game, place significant demands on both anaerobic and aerobic systems. With average VO_2_ max values of approximately 50 mL·kg^−1^·min^−1^ and match workloads corresponding to ~80% VO_2_ max, a high level of aerobic conditioning is necessary for optimal performance and recovery [19]. Enhancing aerobic fitness has also been linked to improvements in technical and tactical execution, as evidenced by increased sprint frequency, ball contacts, and effective game actions [20,21].

The boundaries of the aerobic zone were estimated using the Physical Working Capacity at a heart rate of 170 beats per minute (PWC170) index. Astrand and Ryhming [22] described the PWC170 value as a reliable indicator of aerobic capacity, showing a strong correlation with maximal oxygen uptake. According to Rowland et al. [23] PWC170 demonstrates a correlation of approximately r = 0.70 in predicting VO_2_ max, while Boreham et al. [24] reported a correlation of r = 0.84 in both boys and girls, emphasizing the validity of the PWC170 test in youth populations.

Given these considerations, the purpose of this study is to investigate the intensity-dependent changes of oxygen deficit in elite female handball players aged 14–16 years, using laboratory-based spiroergometric testing. Specifically, we aim to analyze the dynamics of oxygen deficit during maximal exercise and to identify the physiological and morphological factors influencing it. We hypothesized that the oxygen deficit observed within the aerobic zone would impact peak performance indicators. A fundamental aim of the study was to gain insights into the components of cumulative oxygen deficit during sub-threshold (i.e., below the anaerobic threshold) workloads, and to explore the potential for modifying (reducing) these values.

## 2. Materials and Methods

Given the assumption that during workloads up to the PWC170 threshold, the contribution of secondary carbon dioxide (CO_2_) sources is negligible, it appears justified to use the American College of Sports Medicine (ACSM) recommendations to estimate the oxygen deficit. The PWC170 index was treated as a working definition, and the VO_2_ values of the considered stages did not exceed 80% of peak values.

### 2.1. Subjects

Forty-two elite female 14–16-year-old (15.4 ± 1.3 years) handball players were examined in the study. All players were members of the Hungarian handball selection system, with an average of 8 years of training experience. The players regularly played matches according to the rules of the Hungarian competition system. The sample selection was based on the proposal and request of the Hungarian Handball Federation.

Players were informed in advance of the nature of the measurements, their course, and risks, and their parents/guardians gave their consent in writing to do the measurements. The Research Ethics Committee of the Hungarian University of Sports Science (TE-KEB/27/2020) has approved the research.

Before the treadmill loads, contraindications were excluded, and the treadmill tests were performed only after the resting cardiological examinations (standard 12-lead electrocardiogram (ECG), Amedtec ECG Pro, resting heart echo recordings were performed by GE VIVID E95).

### 2.2. Design and Procedures

Participants were enrolled in a one-day study. Body compositions were investigated by the Lunar Prodigy Primo V16 type DEXA instrument [25]. Skeletal muscle mass (kg) was calculated according to Kim et al. [26].

The cardiorespiratory data were collected during a spiroergometry test on a treadmill (Woodway Gmbh, Weil am Rhein, Germany). The intensity of the exercise was increased linearly every minute until maximal oxygen uptake criteria were achieved and subjective signs and symptoms were observed too [27]. The vita maxima protocol was used.

**Figure 1:**
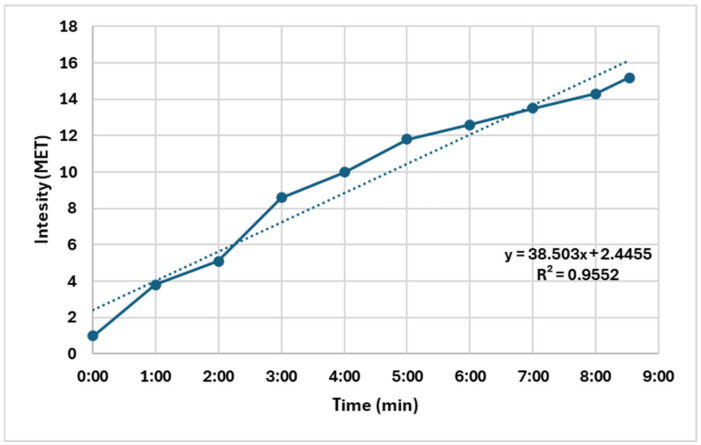
Quasi ramp protocol of a female handball player (body weight: 66 kg) with 1 min stages (the dotted line is the regression line). Note: The MET (Metabolic Rate) shows a near-linear pattern during exercise; the estimated regression has a very strong variance (R = 0.95).

The loading method was adjusted to the Treadmill Ramp Protocol by using simultaneous changes in speed and grade (Figure 1). Based on the assumption and practical observation that trained athletes respond more rapidly to changes in intensity than untrained individuals, the duration of each load stage was reduced to one minute. This also meant in the sample of the studied handball athletes that the total loading time reached or exceeded the generally accepted value of 9 min (the average was 8.5 min) only in athletes providing excellent physical performance. The treadmill protocol used was a ramp-like protocol in which the intensity increased continuously every minute, rather than in fixed-duration stages. All participants followed the same protocol regardless of their cardiorespiratory fitness level. We chose this approach to ensure consistency across participants and to allow for a gradual increase in intensity, which is characteristic of ramp protocols.

The spiroergometric data collection was performed by using Vyare Medical Vyntus CPX gas analyzers (Vyaire Medical GmbH, Hoechberg, Germany), and the Breath-by-Breath (BxB) method. In this method, gas exchange parameters (such as oxygen uptake and carbon dioxide output) are measured for each individual breath in real time. ECG parameters and the most important gas exchange indicators were continuously monitored during the examinations. The spiroergometric data were continuously displayed during each sampling.

Table 1 shows the exercise protocol of a female handball player (body weight: 66 kg). Since her performance, body composition, and gas exchange values were close to the group average, her performance could be assumed to be typical for the studied sample of handball players. Measurements were made in the daily hours (between 10:00 a.m. and 13:00 p.m. The temperature in the laboratory was ~22 degrees Celsius, and the relative humidity was between 25 and 35.

From the data collected during the measurement, the following variables were included in the analysis: maximum time spent on the treadmill: Time max (sec), heart rate: HR (bpm), ventilation: VE (L/min), breath frequency: BF (breaths/min), tidal volume: VT (L), carbon dioxide output: VCO_2_ (mL/min), oxygen uptake: VO_2_ (mL/min), relative oxygen uptake VO_2_rel (mL/min/kg), oxygen pulse (mL).

In addition, maximum power (watt) and maximum power normalized to the body weight (watt/kg) were extracted from the gas analyzer software (SentrySuite 2.17) using the following two formulas (TAN refers to the tangent function) [28]:

In the first load step,

power (watt) = (speed (km/h) × body weight (kg) × (2.05 + 0.29 × TAN (slope%) × 100) − 0.6 × body weight (kg) − 151)/10.5.

In the second load step,

power (watt) = (speed (km/h) × body weight (kg) × (2.11 + 0.25 × TAN (slope%) × 100) − 2.2 × body weight (kg) − 151)/10.5.

It is generally assumed that in trained athletes, physiological responses at a heart rate of 170 bpm do not surpass the anaerobic threshold, typically defined as ≤80% of peak values. This assumption was derived by estimating the oxygen deficit at each workload stage, using the target values recommended by the American College of Sports Medicine (ACSM) [29]. The amount of oxygen required for one minute of exercise intensity was determined using the following equation: VO_2_ (mL/kg) = (0.2 × velocity [m/min]) + (0.9 × velocity [m/min] × grade) + 3.5. These regression estimates are primarily applicable to the aerobic phase of treadmill exercise; therefore, the estimated values were only assigned to workloads below the anaerobic threshold (i.e., <80% of VO_2_ peak). The oxygen deficit value was calculated as the difference between the 1-min oxygen uptake measured in the current load stage and the estimated “required” value. Oxygen deficit values were standardized during the statistical analysis, and a new variable, oxygen deficit/body weight (relO_2_ deficit), was calculated.

By working with the individual data, only data from the same load stages, regardless of the load time (Time max), were considered. Those variables were selected from the measured and calculated variables that were hypothesized, influencing the development of the oxygen deficit.

### 2.3. Statistics

Data were analyzed using SPSS software (Version 19.0; IBM, New York, NY, USA).

Descriptive statistics (mean, median, SE of mean, SD, minimum, and maximum) were calculated for the studied variables. The level of significance was set to 0.05. Pearson correlation was used to analyze the relationship between the continuous variables. Principal Component Analysis (PCA) was used to transform the original correlated variables into a smaller set of orthogonal components, thereby reducing redundancy and facilitating clearer interpretation of latent constructs underlying the dataset. The following variables were included in the analysis: body mass, body height, skeletal muscle mass, fat mass, bone mass, BMD z-score, VO_2_ peak, power peak, VE peak, VCO_2_ peak, O_2_pulse peak, power 170, VCO_2_ 170, VO_2_ 170, oxygen deficit sum, pulse peak, time max, BF peak, VT peak. Hierarchical cluster analysis was used to identify subgroups within the variables. Extraction method was principal component analysis. Hierarchical cluster analysis was used to identify subgroups within the variables. Student *t*-test and the estimation of the standardized effect size (Cohens d) were used for measuring the difference between two subgroups of handball players formed by the results of the cluster and PCA analyses.

## 3. Results

The basic statistics of the variables examined are included in Table 2.

By assuming that the oxygen deficit experienced in the quasi-steady state affected the maximum or peak performances, the relationships among Time max, VO_2_ peak, and O_2_ deficit were studied as a first step. The Pearson correlation was estimated among these variables (Table 3). At first glance, it can be stated that there is a negative relationship between body composition indicators and peak performance. Surprisingly, in the case of muscle mass, this did not bring the expected result, i.e., the higher the muscle mass, the longer the load performance found. The basis of the “shift right” tendency related to training adaptation was the time corresponding to the higher HR = 170, which meant that the later HR = 170 time influenced the performance; the anaerobic threshold (AnT) appeared later. This assumption is confirmed by the observation that a decrease in the VO_2_/kg 170, the smaller O_2_ deficit was observed. The relationship of body composition indicators with O_2_ deficit was positive in all cases; the bigger the body mass components, the bigger the oxygen deficit found. The results of the correlation analyses did not follow the expected pattern. Individuals with higher body weight performed with lower oxygen uptake. Body weight showed a negative correlation with physical performance (Time Max). Since it was surprising that the VO_2_ peak value did not show a significant relationship with exercise duration, we must assume that the effect of the oxygen deficit accumulating in the early stages of exercise cannot be described by linear relationships. By considering the results of the principal component analysis, the role of the studied variables in physical performance can be established. Table 4 shows that the cumulative variance explains 80% of the results. The principal components can be biologically explained. Component 1 explains the factors influencing the VO_2_ peak value. The factor weights confirmed the results seen in the correlation matrix; the absolute oxygen uptake depends on body mass components and gas exchange parameters. The two high-weight values of component 2 were oxygen deficit and HR. The relationship between the maximum running time on the treadmill and body height is a surprise (component 3) is difficult to explain. In component 4, among the respiratory function variables, the fourth principal component strongly correlated with respiratory frequency and, as a new variable, respiratory depth.

It was assumed that applying cluster analysis on the extracted components enables the identification of homogeneous groups of individuals who share similar profiles in terms of body composition, cardiorespiratory function, and metabolic characteristics. This method enhances both the statistical validity and interpretability of the resulting clusters, avoiding spurious groupings that could arise from high dimensional, collinear input data.

This combination of PCA and cluster analysis thus allows for a more robust exploration of latent phenotypes and supports hypothesis generation regarding the physiological determinants of key performance outcomes, such as cumulative oxygen deficit. The results of the cluster analysis are presented in Table 5.

Two distinct clusters were identified based on morphological, metabolic, and performance-related variables (Cluster 1: n = 23; Cluster 2: n = 19). Statistical comparisons revealed both statistically significant and practically meaningful differences across several physiological domains, as indicated by *p*-values and corresponding effect sizes (Cohen’s d).

Pronounced morphological differences were observed between the two clusters. Participants in Cluster 1 exhibited significantly greater body mass (M = 69.67 ± 8.13 kg vs. 59.66 ± 6.81 kg; *p* < 0.001; d = −1.32), skeletal muscle mass (*p* < 0.001; d = −1.21), fat mass (*p* < 0.001; d = −1.32), and bone mass (*p* = 0.034; d = −0.68) compared to Cluster 2. These differences are indicative of a more robust somatotype in Cluster 1.

Despite these advantages in absolute body composition, Cluster 2 demonstrated superior relative aerobic and metabolic performance. Specifically, relative peak oxygen uptake (VO_2_ peak relative to body mass) was significantly higher in Cluster 2 (51.43 ± 3.70 mL·kg^−1^·min^−1^) compared to Cluster 1 (45.70 ± 2.87 mL·kg^−1^·min^−1^; *p* < 0.001; d = 1.76). Similarly, peak power output normalized to body mass was significantly greater in Cluster 2 (*p* < 0.001; d = 1.90), suggesting a higher degree of endurance-related physiological adaptation.

In addition, ventilatory and gas exchange efficiency parameters—specifically ventilation at 170 beats per minute (VE_170_) and carbon dioxide output at the same submaximal heart rate (VCO_2170_)—were significantly lower in Cluster 2. This finding suggests a reduced ventilatory demand and potentially more efficient respiratory control. The substantial effect sizes observed for relative VO_2_ (d = 1.76) and relative VCO_2_ (d = 2.21) further support these interpretations.

Importantly, oxygen deficit corrected for body mass (Deficit_rel) was significantly greater in Cluster 2 (39.03 ± 16.71 mL·kg^−1^) compared to Cluster 1 (32.56 ± 14.33 mL·kg^−1^; *p* = 0.018; d = 0.80). While seemingly paradoxical given the superior aerobic capacity of Cluster 2, this pattern may reflect one or more underlying factors. First, it could be associated with a higher absolute workload at the onset of exercise, potentially due to greater overall fitness levels. Alternatively, it might indicate a delayed activation of oxidative metabolism, suggesting slower VO_2_ kinetics. Finally, the response could result from a disproportionate development of cardiopulmonary capacity relative to the increase in body mass.

This finding underscores that a higher VO_2_ peak does not necessarily equate to a lower oxygen deficit during the early phase of exercise, highlighting the importance of dynamic metabolic responses in addition to steady-state performance metrics.

In summary, the comparative analysis between the two identified clusters revealed clear and meaningful differences across a range of physiological and performance-related parameters. Most notably, the variable Deficit_rel—representing oxygen deficit normalized to body mass—was significantly higher in Cluster 2 despite their superior relative aerobic capacity (VO_2_ peak_rel: 51.43 ± 3.70 vs. 45.70 ± 2.87 mL·kg^−1^·min^−1^; *p* < 0.001; d = 1.76). This suggests a potentially greater reliance on anaerobic energy systems at exercise onset, or a delayed oxidative response, despite higher overall aerobic capacity.

These findings highlight the complexity of early-stage metabolic regulation and suggest that VO_2_ kinetics and initial energy system dynamics may play a more critical role than peak aerobic capacity alone in determining the oxygen deficit during the onset of exercise.

## 4. Discussion and Conclusions

By considering the results of the principal component analysis, it could be stated that the increase in heart rate could indicate the difference between the required and actual values [30]. The question arises as to what kind of training modifications can be used to reduce the early deficit increment. Of the body structural indicators, only the reduction of fat mass is a possible option. The reduction of muscle mass is not justifiable in handball players, extreme fatness can only be seen in the pivot position [31]. Reducing the deficit in the case of gas exchange indicators is conceivable if the oxygen uptake in the steady state phase approaches the required value. The possibilities for this are very limited in some cases. VE depends largely on body dimensions, and its adaptation options are difficult due to the imprinting feature of the breathing pattern (respiratory frequency and depth). The adaptation of oxygen transport, including HR and O_2_ pulse, can be seen in regulation, but also in the respiratory change in volumes. The current research could not address metabolic responses, tissue gas exchange, or the role of oxygen supply.

The cluster analysis revealed significant differences in several anthropometric, cardiovascular, and metabolic variables between athletes with low and high cumulative oxygen deficits. These findings highlight critical factors that may influence oxygen uptake dynamics during submaximal exercise and provide valuable insights into the underlying mechanisms of early fatigue and suboptimal aerobic adaptation. One of the central observations is the role of body mass in shaping theoretical oxygen demand. A higher body mass is associated with a greater absolute oxygen requirement, which, if unmatched by adequate oxygen delivery and utilization, results in a larger oxygen deficit [32]. However, within athletic populations, modifications to body composition are limited in scope. Reductions in skeletal muscle and bone mass are generally undesirable due to their contribution to force production and mechanical support. Furthermore, fat mass in trained individuals typically falls within optimal physiological ranges, minimizing its relevance as a modifiable factor in deficit management [33]. Cardiorespiratory variables also emerged as important determinants. Tidal volume, although dependent on body size, demonstrated limited inter-cluster variability, suggesting that respiratory rate patterns may not significantly contribute to the observed deficits. Instead, genetically influenced cardiovascular parameters such as stroke volume and cardiac output appear to play a more substantial role [34]. These parameters are inherently linked to cardiac structure and function, and beyond a certain point, improvements, especially in ejection fraction may not be attainable through training alone. From a metabolic perspective, the ability to minimize oxygen deficit is largely governed by the oxidative capacity of skeletal muscle tissue. This encompasses mitochondrial density, capillary networks, and oxidative enzyme activity [35]. While endurance training can enhance these properties to a degree, the extent of structural and functional adaptation remains a subject of ongoing debate. Notably, mitochondrial biogenesis and efficiency improvements vary considerably among individuals and are likely constrained by genetic predisposition.

This result highlights that superior aerobic capacity does not necessarily entail lower oxygen deficit per kilogram, especially in the context of higher absolute and relative workload demands. It suggests that VO_2_ kinetics and initial metabolic adjustments may play a more critical role than steady state capacity alone in determining early-stage energy balance [36,37]. Taken together, the findings support the notion that in well-trained individuals, the magnitude of the cumulative oxygen deficit is determined predominantly by intrinsic physiological traits, particularly those that are genetically influenced [38]. This underscores the importance of early life development of cardiorespiratory capacity, as many foundational adaptations, especially those related to cardiac morphology and mitochondrial efficiency, are most responsive during periods of growth and maturation. Although some compensatory adaptation is possible in adulthood, the window for maximal physiological plasticity is limited [39]. These insights provide a foundation for future work aiming to refine individualized training strategies to optimize oxygen kinetics and minimize subthreshold deficits. Further research using longitudinal and intervention-based designs will be necessary to establish causal links and practical recommendations for reducing cumulative oxygen deficit in elite athletes. The findings of this cluster-based analysis highlight the complex interplay between aerobic capacity, body composition, and the dynamic regulation of oxygen uptake during exercise onset. Although Cluster 2 athletes demonstrated superior VO_2_ peak and relative performance metrics, their higher body-mass-corrected oxygen deficit (Deficit rel) suggests a greater initial reliance on anaerobic metabolism.

From a practical standpoint, these findings suggest that optimizing endurance performance requires more than simply increasing VO_2_ peak. Several targeted strategies emerge from the observed associations within the sample. First, athletes with high VO_2_ peak values should aim to improve both aerobic power and VO_2_ kinetics simultaneously. This can be achieved through training modalities such as moderate to high-intensity intervals, which help accelerate VO_2_ kinetics and minimize the oxygen deficit during the initial phase of exercise [40]. Second, enhancing movement efficiency and power economy is essential. High peak power outputs (e.g., peak watts per kilogram) should be complemented with submaximal work efficiency training to prevent excessive reliance on anaerobic pathways during exercise initiation [41]. Furthermore, a balance must be maintained between muscle mass and aerobic capacity. While increased skeletal muscle mass supports force production, it must be matched by adequate aerobic adaptation to avoid an unsustainable rise in oxygen demand [42]. In addition, optimizing body composition—particularly by reducing nonfunctional mass such as excess adipose tissue—can lower the oxygen cost per unit of body weight and consequently improve relative oxygen deficit (Deficit rel) [43]. Finally, improving ventilatory efficiency is another key consideration. Elevated VE170 and VCO_2_ values may indicate inefficiency in the respiratory system. Targeted interventions, including breathing pattern optimization, respiratory muscle training, and sustained submaximal exercise, may facilitate more effective early-stage aerobic activation [44].

These insights reinforce the value of incorporating dynamic oxygen deficit metrics like Deficit rel into performance diagnostics, particularly in endurance sports. This measure can guide individualized training plans aimed at minimizing anaerobic load during critical phases of competition or effort onset.

**Limitations of the study:**

The present study should be interpreted within the specific context of elite handball performance. In recent years, the relative contribution of the body’s energy systems in handball has evolved, with an observable shift toward increased reliance on anaerobic metabolism due to the higher tempo and intensity of the modern game. Although the aerobic system remains fundamental as a base, the growing role of anaerobic pathways has significant implications for training methodology and athlete development.

Therefore, while the findings on oxygen deficit and its determinants (e.g., VO_2_ kinetics and body composition) are grounded in general physiological principles, their direct applicability to pure endurance sports (e.g., long-distance running, and cycling) should be considered with caution. The kinetics of oxygen deficit during submaximal and maximal loading represent a universal physiological response, but the magnitude and functional relevance of influencing factors may vary significantly across sports disciplines.

These limitations underscore the importance of sport-specific interpretation of physiological diagnostics and support the need for tailored performance assessment models across different training and competition profiles.

## Figures and Tables

**Table 1:** Spiroergometric loading protocol of a female handball player (body weight: 66 kg). Yellow cells: the oxygen deficit was calculated for these stages.

Stage	Time in Stage (min:s)	Time in Exercise (min:s)	Speed (km/h)	Grade (%)	METs	HR (bpm)
Resting	2:01	0:00	0.0	0.0	1	96
Stage1	1:00	1:00	6.0	0.0	3.8	114
Stage2	1:00	2:00	6.0	2.5	5.1	121
Stage3	1:00	3:00	7.5	3.0	8.6	146
Stage4	1:00	4:00	8.3	3.0	10	155
Stage5	1:00	5:00	10.0	3.0	11.8	170
Stage6	1:00	6:00	10.0	5.0	12.6	176
Stage7	1:00	7:00	10.0	7.0	13.5	182
Stage8	1:00	8:00	10.0	9.0	14.3	188
Stage9	0:32	8:32	10.0	11.0	15.2	189
Recovery	5:03	0:00	0.0	0.0	1	101

**Table 2:** Basic statistics of the studied variables of athletes, N = 42.

	Mean	Median	SE of Mean	SD	Min	Max
**Body Composition**						
Body height (cm)	172.48	172.00	0.86	5.56	161.00	186.00
Body mass (kg)	65.14	64.40	1.39	9.01	48.00	88.80
Skeletal muscle mass (kg)	23.77	24.08	0.49	3.18	18.46	32.22
Fat content (kg)	17.76	17.36	0.69	4.49	9.40	28.46
Mineral content(kg)	2.68	2.67	0.05	0.30	2.12	3.50
Absolut Z-score	2.29	2.4	0.15	0.95	0.3	4.1
**Gas exchange values**						
VE peak (L/min)	93.89	91.23	1.82	11.76	76.14	121.24
BF peak (breath/min)	49.66	49.7	1.15	7.46	35.8	67.2
VT peak (L)	2.05	2.01	0.06	0.37	1.4	3.12
VO_2_ peak (L/min)	3.13	3.04	0.06	0.37	2.52	3.85
VCO_2_ peak (L/min)	3.35	3.26	0.06	0.38	2.73	4.06
O_2_pulse peak (mL)	16.44	16.14	0.43	2.78	12.61	28.97
HR peak (beat/min)	192.81	193	1.22	7.90	177	210
**In-range quasi-aerobe zone**						
VE 170 (L/min)	58.21	56.49	1.85	12.00	39.55	95.3
VO_2_ 170 (L/min)	2.29	2.17	0.06	0.38	1.70	3.28
VCO_2_ 170 (L/min)	2.08	2.00	0.06	0.37	1.52	3.20
**Physical performance**						
Power max (watt)	285.40	277.48	5.29	34.30	227.55	367.09
Power 170 (watt)	167.14	158.11	5.51	35.73	118.96	272.06
Time max (sec)	512.71	511	7.15	46.35	417.00	660
**Estimated oxygen deficit**						
Deficit sum of stage 1–6 (L)	2.32	2.32	0.17	1.08	0.69	4.63
Deficit sum/weight (L/kg)	35.49	34.32	2.40	15.60	9.84	65.73

VE: ventilation, BF: breath frequency, VT: tidal volume, VO_2_: maximal oxygen uptake, VCO_2_: carbon dioxide output, O_2_pulse: oxygen pulse, Time max: maximum time spent on the treadmill.

**Table 3:** Pearson correlation coefficients (r) and *p* values (*p*) of Time max, oxygen deficit, and VO_2_ peak with the studied body structural and spirometry parameters (bold correlation coefficients are significant at 0.05 level).

Variables	Time Max		O_2_ Deficit		VO_2_ Peak	
	r	*p*	r	*p*	r	*p*
Body height	**−0.456**	0.002	0.108	0.498	**−0.466**	0.002
Body mass	**−0.565**	<0.001	**0.329**	0.033	**−0.518**	<0.001
VO_2_ peak	0.220	0.264	0.225	0.152	-	-
Skeletal muscle mass	**−0.527**	<0.001	**0.340**	0.028	**−0.561**	<0.001
Bone mass	**−0.389**	0.011	**0.425**	0.005	**−0.326**	0.035
Z-score (BMD)	**−0.368**	0.017	**0.448**	0.003	−0.224	0.154
Fat mass	**−0.571**	<0.001	0.292	0.089	**−0.449**	0.035
Power peak	0.003	0.983	**0.361**	0.019	−0.180	0.255
HR peak	0.082	0.605	**0.384**	0.012	0.034	0.829
VE peak	0.001	0.995	0.082	0.606	0.049	0.758
BF peak	0.147	0.354	−0.299	0.055	0.193	0.220
VT peak	−0.213	0.176	**0.361**	0.017	−0.214	0.173
VCO_2_ peak	−0.035	0.826	0.265	0.090	0.054	0.722
O_2_pulse peak	−0.107	0.499	0.075	0.636	0.101	0.525
Power 170	−0.042	0.786	0.083	0.601	−0.143	0.302
VE 170	−0.261	0.096	−0.102	0.519	−0.157	0.321
VCO_2_ 170	−0.234	0.135	−0.042	0.794	−0.162	0.306
VO_2_ 170	−0.293	0.060	0.030	0.852	−0.099	0.533

VE: ventilation, BF: breath frequency, VT. tidal volume, VO_2_: maximal oxygen uptake, VCO_2_: carbon dioxide output, O_2_pulse: oxygen pulse.

**Table 4:** Principal component analysis (bold correlation coefficients are significant at 0.05 level).

	Components			
Variables	PC1	PC2	PC3	PC4
VO_2_ peak (L/min)	**0.873**	−0.018	0.279	0.092
Body mass (kg)	**0.925**	0.194	−0.145	0.226
Skeletal muscle mass (kg)	**0.829**	0.187	−0.179	0.197
Fat mass (kg)	**0.848**	0.202	−0.118	0.326
Power peak (watt)	**0.874**	0.028	0.308	−0.034
VE peak (L/min)	**0.770**	−0.322	0.320	0.010
VCO_2_ peak (L/min)	**0.846**	0.002	0.336	−0.084
O_2_pulse peak (mL)	**0.825**	−0.280	0.214	−0.019
Power 170 (watt)	**0.749**	−0.516	−0.054	−0.206
VCO_2_170 (L/min)	**0.775**	−0.481	−0.217	−0.159
VO_2_ 170 (L/min)	**0.869**	−0.367	−0.151	−0.050
Bone mass (kg)	**0.860**	0.290	0.032	−0.002
BMD z-score	**0.618**	0.485	0.216	0.161
Deficit sum (L)	0.306	**0.607**	0.216	−0.111
HR peak (beat/min)	−0.203	**0.785**	0.379	0.164
Time max (sec)	0.362	−0.297	**0.696**	−0.441
Body heigh (cm)t	0.467	0.098	**−0.578**	−0.040
BF peak (breath/min)	−0.163	−0.589	0.301	**0.674**
VT peak (L)	0.598	0.361	−0.126	**−0.601**
eigenvalue	9.685	2.808	1.738	1.359
variance	51.0%	14.8%	9.1%	7.2%
KMO and Bartlett’s test	*p* < 0.001			

VE: ventilation, BF: breath frequency, VT: tidal volume, VO_2_: maximal oxygen uptake, VCO_2_: carbon dioxide output, O_2_pulse: oxygen pulse, Time max: maximum time spent on the treadmill.

**Table 5:** Differences between the low and high oxygen deficit clusters.

	QCL_5 Cluster Number of Case	N	Mean	Std. Deviation	Std. Error Mean	*p t*-Test	Cohen’s d
Body mass (kg)	1	23	69.665	8.1329	1.6958	<0.001	−1.3220
	2	19	59.663	6.8082	1.5619		
Body height (cm)	1	23	174.3	5.406	1.127	0.017	−0.7716
	2	19	170.26	5.02	1.152		
Skeletal muscle mass (kg)	1	23	25.2679	2.890406	0.60269	<0.001	−1.2077
	2	19	21.95587	2.549884	0.58498		
Fat mass (kg)	1	23	20014.39	4168.203	869.13	<0.001	−1.3220
	2	19	15026.21	3225.097	739.888		
Bone mass (kg)	1	23	2767.7	314.469	65.571	0.034	−0.6810
	2	19	2569.89	258.081	59.208		
Time max (sec)	1	23	483.09	31.311	6.529	<0.001	1.9870
	2	19	548.58	34.867	7.999		
VO_2_ peak rel (L/min/kg)	1	23	45.69936	2.867183	0.59785	<0.001	1.7551
	2	19	51.43335	3.697539	0.84827		
Power peak (watt)	1	23	4.183721	0.245287	0.05115	<0.001	1.9047
	2	19	4.674177	0.271671	0.06233		
HR peak (beat/min)	1	23	190.09	7.621	1.589	0.012	0.8152
	2	19	196.11	7.086	1.626		
Power 170 (watt)	1	23	178.7716	40.65081	8.47628	0.018	−0.7629
	2	19	153.0498	22.49804	5.16141		
VE 170 (L/min)	1	23	63.2804	12.83398	2.67607	0.002	−1.0457
	2	19	52.07173	7.349767	1.68615		
VCO_2_ 170 (L/min)	1	23	2227.189	393.5583	82.0626	0.004	−0.9544
	2	19	1903.858	256.366	58.8144		
Time max (sec)	1	23	472.78	44.184	9.213	0.002	1.0354
	2	18	513	31.096	7.329		
Deficit rel (L/kg)	1	23	32.5625	14.33114	2.98825	0.0184	0.8028
	2	19	39.03	16.71429	3.83452		
VO_2_ rel (L/min/kg)	1	23	45.6994	2.86718	0.59785	<0.001	1.7551
	2	19	51.4333	3.69754	0.84827		
VCO_2_ rel (L/min/kg)	1	23	48.3992	3.07209	0.64057	<0.001	2.2055
	2	19	55.9041	3.76785	0.8644		

VE: ventilation, Deficit rel: oxygen deficit, VO_2_: maximal oxygen uptake, VCO_2_: carbon dioxide output, Time max: maximum time spent on the treadmill.

## Data Availability

The dataset of the present study is available from the Corresponding Author on reasonable request.
